# Childhood Trauma and Problematic Internet Use Among Sudanese Medical Students 2025: A Cross-Sectional Study

**DOI:** 10.1192/bjo.2026.11062

**Published:** 2026-06-30

**Authors:** Abrar Hashim Alrofaie Sayed, Yousef Mohamed Ahmed Alsalawy, Muzan Ibrahem Issa Alnour, Sohaib Mohammed Mokhtar Ahmed, Danya Ibrahim

**Affiliations:** 1 Faculty of Medicine, University of Khartoum, Khartoum, Sudan; 2 Faculty of Medicine and Health Sciences, University of Gadarif., Gadarif, Sudan; 3 MBBS Faculty of Medicine, University of Khartoum, Khartoum, Sudan

## Abstract

**Aims:**

This study aimed to estimate the prevalence of Childhood trauma (CT) subtypes and problematic internet use (PIU) among Sudanese medical students and the association between them. It examined which subtypes of the CT are most significantly associated with PIU, it also assessed the demographic predictors for PIU.

**Methods:**

A descriptive cross-sectional study was conducted using an online questionnaire, it was distributed among 430 medical students from six universities located in Khartoum and Al Jazeera states, Sudan. Childhood maltreatment was measured using the Childhood Trauma Questionnaire-Short Form (CTQ-SF), and problematic internet use levels were assessed using the Chinese Internet Addiction Scale-Revised (CIAS-R). Both instruments are internationally validated. Descriptive statistics were reported as frequencies and percentages for categorical variables, and as medians with interquartile ranges for non-normally distributed continuous variables; Spearman correlation and multiple linear regression were used to assess associations and identify predictors of PIU while controlling for trauma-related anddemographic variables. A significance level of p <0.05 was applied.

**Results:**

Emotional abuse appeared as the most common form of childhood trauma, 48.8% (n=210), followed by physical neglect, 40.5% (n=174), and sexual abuse, 34.0% (n=146).The prevalence of problematic internet use among the participants was 46.0% (n=198). Also, a significant positive association was observed between PIU and overall CT scores (ρ=0.189, p<0.001). Among childhood trauma subtypes, emotional abuse had the strongest association with problematic internet use (ρ=0.219, p<0.001), while physical neglect, emotional neglect, and physical abuse also contributed. Furthermore, fifth-year students had remarkably lower CIAS-R scores compared to first-year students.

**Conclusion:**

Emotional abuse emerged as the strongest indicator of problematic internet use severity among medical students. These findings emphasize the necessity for trauma-informed interventions, including counselling services and awareness programmes, to indicate the influences of childhood trauma and minimize problematic internet use among Sudanese medical student populations. Future studies should assess the internal psychological factors through which emotional abuse influences problematic internet use; understanding these details will contribute to building more targeted interventions.

